# Retraction: Chronic Epipharyngitis Treated with Epipharyngeal Abrasion Therapy: Symptoms, Diagnosis, Pathogenesis, and Treatment Outcomes

**DOI:** 10.31662/jmaj.R-0001

**Published:** 2025-04-22

**Authors:** Yasuaki Harabuchi, Takumi Kumai, Kensuke Nishi, Ayaki Tanaka, Osamu Hotta, Hitoshi Hagino, Toshiyuki Kusuyama, Manabu Mogitate, Yoshihiro Ohno, Akira Sakakibara, Satsuki Araki, Yoshinao Nishida, Tomoko Shintani, Hiroyuki Takezawa, Hirofumi Ito, Daigo Komazawa, Noriko Nishiwaki, Ryuzo Toritani, Koichi Hirahata, Satoshi Marumo

**Affiliations:** 1 Epipharyngeal Abrasion Therapy Review Committee, Japan Society of Stomato-pharyngology, Tokyo, Japan; 2 Japanese Focal Inflammation Related Disease Research Group, Sendai, Japan; 3 Department of Otolaryngology-Head and Neck Surgery, Asahikawa Medical University, Asahikawa, Japan; 4 Department of Otolaryngology, Fukuoka Dental College, Fukuoka, Japan; 5 Tanaka Otorhinolaryngology Clinic, Osaka, Japan; 6 Hotta Osamu Clinic, Sendai, Japan; 7 Hagino Otolaryngology Clinic, Tokyo, Japan; 8 Tokyo Voice Clinic, Tokyo, Japan; 9 Mogitate Otorhinolaryngology Clinic, Kawasaki, Japan; 10 Ohno Otorhinolaryngology Clinic, Tokyo, Japan; 11 Asahi-cho Sakakibara Otorhinolaryngology Clinic, Yamagata, Japan; 12 Araki Otorhinolaryngology Clinic, Fukuroi, Japan; 13 Nishida Otorhinolaryngology Clinic, Toyonaka, Japan; 14 Tomo Otolaryngology Clinic, Sapporo, Japan; 15 Takezawa Otorhinolaryngology Clinic, Obihiro, Japan; 16 Ito Otorhinolaryngology Clinic, Funabashi, Japan; 17 Akasaka Voice Health Center, Tokyo, Japan; 18 Motohashi Otorhinolaryngology Clinic, Mishima, Japan; 19 Toritani Clinic, Kumamoto, Japan; 20 Hirahata Clinic, Tokyo, Japan; 21 Respiratory Organ Center, Kitano Hospital, Osaka, Japan

Retraction: Harabuchi Y, Kumai T, Nishi K, et al. Chronic epipharyngitis treated with epipharyngeal abrasion therapy: symptoms, diagnosis, pathogenesis, and treatment outcomes. JMA J. 2025;8(2):371-384.

The above article is available at https://www.jmaj.jp/detail.php?id=10.31662%2Fjmaj.2024-0437.

The above article, published online on March 21, 2025, in JMA Journal, has been retracted.

A third party pointed out that the article was a duplicate publication of previously published article. Then, the authors contacted the Support Office. The editor-in-chief has therefore decided to retract the article.

